# Antioxidant supplementation in hypertension

**DOI:** 10.12861/jrip.2014.13

**Published:** 2013-11-03

**Authors:** Mohammad Reza Ardalan, Mahmoud Rafieian-Kopaei

**Affiliations:** ^1^Chronic Kidney Disease Research Center, Tabriz University of Medical Sciences, Tabriz, Iran; ^2^Medical Plants Research Center, Shahrekord University of Medical Sciences, Shahrekord, Iran

**Keywords:** Oxidative stress, Inflammation, Hypertension, Antioxidants

**Implication for health policy/practice/research/medical education:**

Hypertension is considered as a major risk of cardiovascular diseases worldwide. It has been evidenced that oxidative stress is increased in patients with salt-sensitive hypertension, essential hypertension, malignant hypertension, renovascular hypertension, preeclampsia as well as cyclosporine-induced hypertension. Plants and animals have complex systems of multiple types of antioxidants, including glutathione, vitamins A, C, and E, and enzymes like superoxide dismutase, catalase, and several peroxidases. The diets rich in fruits and vegetables can reduce blood pressure in hypertensive and normotensive patients.

Cardiovascular disease is accompanied with enhanced oxidative stress (OS) in the vascular wall, heart, kidney, and brain. OS contributes to vascular injury by promoting inflammation, endothelial dysfunction and increased vascular tone, leading to altered vascular contractility, structural remodeling, and hypertension as well as other forms of cardiovascular disease. Hypertension is considered as a major risk of cardiovascular diseases worldwide. It has been evidenced that OS is increased in patients with salt-sensitive hypertension, essential hypertension, malignant hypertension, renovascular hypertension, preeclampsia as well as cyclosporine-induced hypertension (1). The mechanisms involved in generation of excess reactive oxygen species (ROS) and the mechanisms interfering with antioxidant defense in hypertension are not clear, too. Diminished antioxidant defense stems from deficiencies in glutathione peroxidase, catalase or superoxide dismutase. Attempts to target ROS are based on reducing ROS generation and increasing antioxidant bioavailability (2). Although excessive ROS are central pathway in induction and exacerbation of hypertension, however, there are controversies about the efficacy of antioxidant consumption in hypertension therapy (3). Some studies suggest that diets with high antioxidants content may reduce blood pressure and cardiovascular complications. Furthermore, it has been hypothesized that there might be a correlation between stress oxidative and arterial hypertension. In contrast, some randomized clinical and population studies have shown disappointing results. The variety in the results of different studies has been attributed to variety in trial design or dosing regimens. However, the potential pro-oxidant activity of antioxidants is a phenomenon which should not be ignored. It is generally recommended that general population, particularly the patients with OS induced complications should consume a balanced diet with emphasis on antioxidant-rich vegetables and fruits and whole grains (4). Antioxidants inhibit molecules’ oxidation which, in turn, produces free radicals capable of starting chain reactions. When the chain reaction starts in a cell, it could damage the cell or cause its death. By removing free radicals, antioxidants terminate these chain reactions and inhibit other oxidation reactions by being oxidized (5). Plants and animals have complex systems of multiple types of antioxidants, including glutathione, vitamins A, C, E, as well as enzymes like superoxide dismutase, catalase, and several peroxidases (6,7). The diets rich in fruits and vegetables can reduce blood pressure in hypertensive and normotensive patients (8). In a study, increase in fruit and vegetable intake in the diet of hypertensive participants for 6 months led to increase in blood antioxidant capacity and decrease in systolic and diastolic blood pressure (9). Although these reports support the beneficial effect of dietary antioxidants on hypertension and cardiovascular risk, antioxidant supplementation has not been shown to be effective (10). A study reported that blood pressure did not improve after treatment with a mixture of vitamin C, vitamin E, and beta-carotene versus placebo after 5 years in the participants who were thought to be at high risk of cardiovascular disease (11). Furthermore, a meta-analysis revealed no improvement in cardiovascular-associated mortalities after antioxidant supplementation (12). According to the literature, we could conclude that, the diets high in antioxidants such as fruits and vegetables can reduce blood pressure and cardiovascular diseases, but this is not the case for diet supplementations (13). The possible reason is that, in the diet, there are various antioxidants working as a continuous chain, while supplementation is usually given using one or two substances. Therefore, the antioxidant chain is not completely available. It is also obvious that if an antioxidant, after scavenging free radicals, is not restored by the following antioxidant in the chain, it will gradually turn into a pro-oxidant. As a result, the ultimate effect of such supplementations would be no or a damaging effect (14,15).

## Authors’ contributions

All authors wrote the manuscript equally.

## Conflict of interests

The author declared no competing interests.

## Ethical considerations

Ethical issues (including plagiarism, data fabrication, double publication) have been completely observed by the author.

## Funding/Support

None.
